# Detection of rare variant effects in association studies: extreme values, iterative regression, and a hybrid approach

**DOI:** 10.1186/1753-6561-5-S9-S112

**Published:** 2011-11-29

**Authors:** Zhaogong Zhang, Qiuying Sha, Xinli Wang, Shuanglin Zhang

**Affiliations:** 1 Department of Mathematical Sciences, Michigan Technological University, Houghton, MI 49931, USA; 2 School of Computer Science and Technology, Heilongjiang University, Harbin 150080, China; 3 School of Technology, Michigan Technological University, Houghton, MI 49931, USA

## Abstract

We develop statistical methods for detecting rare variants that are associated with quantitative traits. We propose two strategies and their combination for this purpose: the iterative regression strategy and the extreme values strategy. In the iterative regression strategy, we use iterative regression on residuals and a multimarker association test to identify a group of significant variants. In the extreme values strategy, we use individuals with extreme trait values to select candidate genes and then test only these candidate genes. These two strategies are integrated into a hybrid approach through a weighting technology. We apply the proposed methods to analyze the Genetic Analysis Workshop 17 data set. The results show that the hybrid approach is the most powerful approach. Using the hybrid approach, the average power to detect causal genes for Q1 is about 40% and the powers to detect *FLT1* and *KDR* are 100% and 68% for Q1, respectively. The powers to detect *VNN3* and *BCHE* are 34% and 30% for Q2, respectively.

## Background

Evidence is increasingly showing that complex diseases are caused by both common and rare variants [1-3]. Statistical methods to detect common variants have been well developed. However, these methods are not optimal for detecting rare variants. Recently, several methods have been proposed to detect rare variants, including the combined multivariate and collapsing (CMC) method [4] the Markov chain (MC) method [5], the cohort allelic sums test (CAST) [6], and its weighted version, the weighted-sum (WS) method [7]. These methods essentially test one gene at a time. Because complex diseases are caused by many genes, the existing methods may lose power.

In this paper, we develop two strategies to search for both common and rare variants in multiple genes: iterative regression and extreme values. A hybrid approach of these two strategies is also explored to improve power. In the iterative regression strategy, common single-nucleotide polymorphisms (SNPs) and rare variant combinations are tested first. Then a best variant is selected. The regression is repeated against the residual to discover potential variants. A score test [8] is used for all the selected SNPs to determine whether we should continue the iterative process. In the extreme values strategy, we use the individuals with the top 5% value of the quantitative trait to select candidate genes and then use the score test [8] to test each candidate gene. We apply the proposed methods to the Genetic Analysis Workshop 17 (GAW17) data set to detect genes that are associated with two quantitative traits.

## Methods

### Data preparation

The variants of the GAW17 data set are divided into common and rare. We define a variant as rare if its minor allele frequency (MAF) is less than 0.01. Within each gene, we collapse all rare variants to obtain a rare variant combination (RVC) [4]. For an RVC, we code the genotype for the *i*th individual as 1 if the *i*th individual has at least one rare mutation within the RVC; otherwise the genotype is coded as 0. For a common SNP with two alleles *a* and *A*, we define the numerical code of genotype for the *i*th individual as *x_i_* = 0, 1, or 2 for genotype *aa*, *aA*, or *AA*, respectively. The GAW17 data set contains genotypes at 24,487 SNPs in 3,205 genes on chromosomes 1–22 with 209 case subjects and 488 control subjects. In this study, the genes are removed from the data set if they do not have nonsynonymous SNPs. After the removal of 1,009 genes from the data set, 2,196 genes are used for the analysis in the following step. Then, synonymous SNPs in the remaining 2,196 genes are deleted. The original GAW17 data set is transformed into a new data set *G*, which includes 4,711 common SNPs or RVCs.

### Iterative regression strategy

We propose the iterative regression strategy to identify a group of significant common SNPs or RVCs. For this method, we need a multimarker test. We propose to use the score test given by Chapman et al. [8]. Let *x_i_* = (*x_i_*_1_, …, *x_ik_*)*^T^*, and let *y_i_* denote the numerical code of the multimarker genotype and the trait value of the *i*th individual, where *i* = 1, …, *N* and *N* is the sample size. To test the null hypothesis of no association between the *k* markers and the trait, we use the score test statistic given by:(1)

where:(2)(3)(4)

and:(5)

The score test statistic *S* asymptotically follows a chi-square distribution with *k* degrees of freedom.

For a given marker cutoff value *L* and a significance level *α*, the algorithm includes the following steps:

**Step 1.** Use an *L* step procedure (step 11, step 12, …, step 1*L*) to select *L* candidate SNPs or RVCs, denoted *v*_1_, …, *v_L_*. In step 1*j*, a SNP or RVC that has the highest correlation with trait values is selected and denoted *v_j_*. Then, we update the trait value by residual:(6)

where  and  are the least-squares estimators of *β*_0_ and *β*_1_ in the linear model:(7)

**Step 2.** Let *A*_1_, …, *A_L_* denote the *L* candidate SNP sets, where *A_i_* = {*v*_1_, …, *v_i_*}. The score test is used to test association of each *A_i_*. The raw *p-*value *p_i_* is calculated by using a chi-square distribution and the adjusted *p*-value *q_i_* is computed by using a permutation test. The SNP set with the smallest adjusted *p*-value is the final candidate SNP set. The final candidate SNP set is denoted *A_f_*.

**Step 3.** Use a permutation test to evaluate the overall *p*-value of *A_f_*. Denote the overall *p*-value as *p*_overall_. If *p*_overall_ <*α*, the final significant SNP set is the final candidate SNP set *A_f_*. Otherwise, the final significant SNP set is empty.

Using a standard permutation procedure, we obtain *p*_overall_ through another layer of permutation. We use a permutation procedure recently proposed by Ge et al. [9] to evaluate adjusted *p*-values and the overall *p*-value at the same time using one layer of permutation. The permutation procedure includes the following steps:

**Step 1.** Generate *S* (say, 1,000) permuted data sets. In each permutation, we randomly shuffle trait values. For each permuted data set, search for the *L* candidate SNP sets by using the same procedure. For the *s*th permuted data set (the 0th data set is the real data set), denote the *L* candidate SNP sets by *A_s_*_1_, …, *A_sL_* and the associated raw *p*-values by *P_s_*_1_, …, *P_sL_*. Then, the adjusted *p*-value corresponding to the candidate SNP set *A_l_* is estimated by:(8)

where *I*(·) is the indicator function. We choose the SNP set with the smallest adjusted *p*-value,(9)

as the final candidate SNP set.

**Step 2.** To evaluate the overall *p*-value of the final candidate SNP set, we first adjust the raw *p*-values *P_s_*_1_, …, *P_sL_* for the *s*th permuted data, *s* = 1, …, *S*. The adjusted value of *P_sl_* is given by:(10)

Let:(11)

Then, the overall *p*-value of the final candidate SNP set is given by:(12)

### Extreme values strategy

Intuitively, for a quantitative trait that has a positive relation with a disease, an individual with more causal mutations will have a higher trait value. Therefore we propose to use an extreme values strategy to select candidate genes for testing association with rare variants.

The extreme values strategy includes two steps.

**Step 1.** We select candidate genes using individuals with extreme trait values (top 5% in this study). In detail, we first select a group of individuals with extreme trait values and denote this group of individuals by *EX* = {*i*: *y_i_* >*C*}. For a gene with an RVC, let *x_i_* denote the numerical code of genotype of the *i*th individual. Then, we define this gene as candidate gene if . In other words, we define a gene as a candidate gene if at least one individual in *EX* has at least one rare mutation within the RVC of this gene.

**Step 2.** We apply the score test to each of the candidate genes. For a given significance level *α*, a candidate gene is declared significant if the *p*-value is less than *α*/*n*, where *n* is the number of candidate genes.

### Hybrid approach

The iterative regression and extreme values strategies are different. One is a gene-based method and the other is a SNP-based method. The integration of the two methods may improve the power to detect association. Thus we propose a hybrid approach. The hybrid approach ends with a group of significant genes and SNPs or RVCs. For a given significance level *α*, let *A_α_* denote the group of significant SNPs provided by the iterative regression strategy and *B_α_* denote the group of significant genes provided by the extreme values strategy. Then, the hybrid approach ends with the union of *A*_*α*/2_ and *B*_*α*/2_.

## Results

We use the three proposed methods and the CMC method to analyze the GAW17 data set. Before the data analysis, we already knew the answers. The first step of data analysis is to adjust trait values for covariates by assuming the linear model:(13)

where *y* is the trait value and *x*_1_, …, *x_k_* are the covariates. In this application, we consider Age, Sex, and Smoking history as covariates. In the following discussion, we use residuals as trait values.

**Figure 1:**
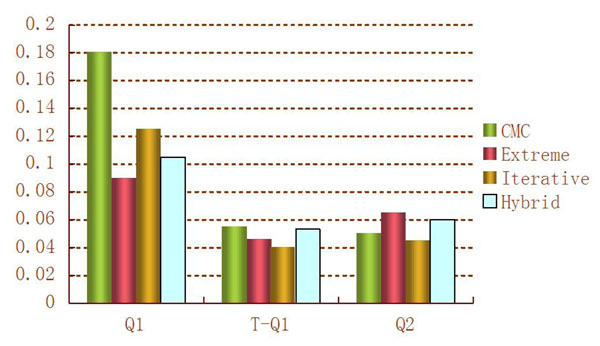
**Type I errors of the four methods** Type I error at a nominal level of 0.05. T-Q1 denotes the value of Q1 after ranking and an inverse normal transformation.

**Figure 2:**
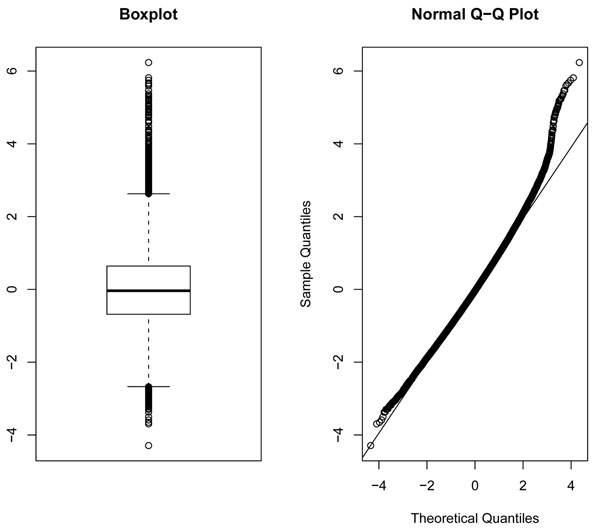
Boxplot and Q-Q plot of Q1

For evaluating the performance of the four methods, we first consider type I error rates. To evaluate type I error rates, we permute trait values in each of the 200 replications. The estimated type I error rates of the four methods based on permuted trait values are given in Figure 1. For 200 replicated samples, the standard deviation of type I error rates is [0.05(0.95)/200]^1/2^ ≈ 0.015, and the 95% confidence interval (CI) is (0.02, 0.08) for the nominal level of 0.05. From Figure 1, we can see that type I error rates for all four methods are within the 95% CI for Q2, whereas type I error rates for all four methods are significantly higher than the nominal level of 0.05 for Q1. This is because of the inflated type I errors caused by outliers, which is depicted in the boxplot and normal quantile-quantile (Q-Q) plot for Q1 shown in Figure 2. To delete the effect of outliers, we rank Q1 and apply an inverse normal transformation. Based on the transformed Q1, type I error rates for all four methods are within the 95% CI (Figure 1). In the power comparisons, we use transformed Q1.

**Figure 3:**
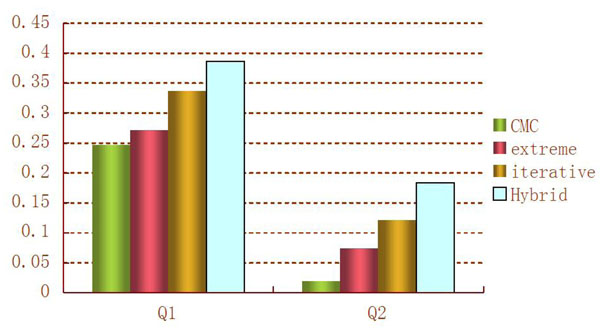
Power of the four methods

**Figure 4:**
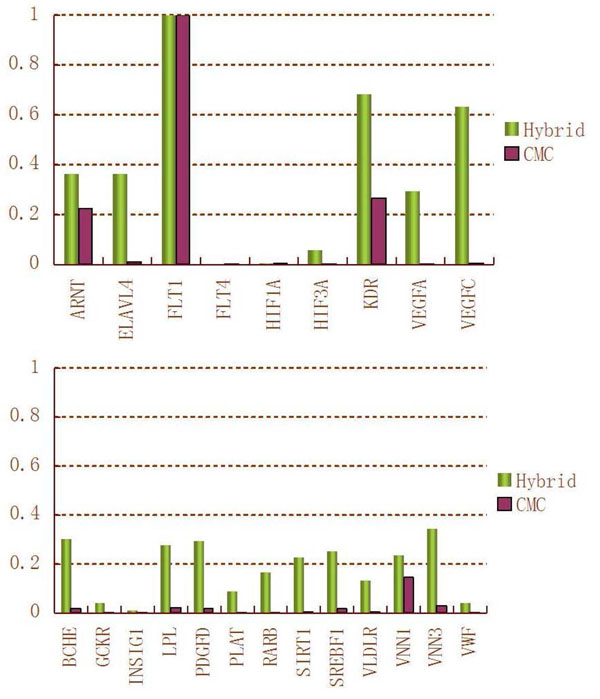
**Powers to detect causal genes using the hybrid and CMC methods** Power for (a) Q1 and (b) Q2.

Quantitative trait Q1 is influenced by 9 genes, and Q2 is affected by 13 genes. For the purpose of power comparisons, we calculate the average power to detect the 9 causal genes of Q1 (called power for Q1) and the average power to detect the 13 causal genes of Q2 (called power for Q2). The power for Q1 and the power for Q2 of the four methods are summarized in Figure 3. This figure shows that the patterns of power comparison for Q1 and Q2 are consistent; that is, from the most powerful to the least powerful, the methods are the hybrid approach, the iterative regression strategy, the extreme values strategy, and the CMC method. This pattern is not hard to understand because the hybrid and iterative regression approaches can consider multiple genes simultaneously, whereas the extreme values and CMC methods consider one gene at a time. We further compare power of the hybrid approach and the CMC method by evaluating the power of detecting each of the causal genes of Q1 and Q2 (Figure 4). We learn from Figure 4 that the hybrid approach is consistently more powerful than the CMC method for detecting the 22 causal genes.

## Discussion

New sequencing technologies that allow researchers to sequence parts of the genome—or, in the future, the whole genome—of large groups of individuals have made rare variant association studies feasible. However, statistical methods to test association between rare variants and phenotypes are still underdeveloped. Existing methods that essentially test one gene at a time may lose power to detect complex disease genes because complex diseases are presumed to be caused by many genes. In this paper, we have developed three novel methods: the iterative regression strategy, the extreme values strategy, and the hybrid approach. The iterative regression strategy can test multiple genes simultaneously, whereas the extreme values strategy can delete less important genes and thus makes the problem of multiple testing less severe. The hybrid approach is the combination of the two strategies. Analysis using the GAW17 data set shows that all three proposed methods are more powerful than the CMC method, one typical existing method used to test rare variant association. In this study, we collapse all rare variants within one gene into a single variant and analyze this variant together with common variants. We can also use other collapsing methods, such as the weighted-sum method [7], to collapse both rare and common variants within one gene into a single variant and apply our methods to the collapsed variants. One problem left for the iterative regression method is choosing an appropriate marker cutoff value *L* (number of candidate SNPs in step 1). If *L* is too small, the iterative regression method may lose power because it cannot include all causal genes. If *L* is too large, the iterative method may also lose power because noise terms are included. Further investigation is needed for choosing the optimal value of *L*.

## Conclusions

We propose three methods for detecting both rare and common variants. Application to the GAW17 data set shows that all three proposed methods are more powerful than the CMC method, one typical existing method used to test rare variant association.

## Competing interests

The authors declare that there are no competing interests.

## Authors’ contributions

ZZ performed all computing, proposed part of the methods, and drafted the manuscript. QS participated in the methodology development and drafted the manuscript. XW helped to draft the manuscript and helped for the methodology development. SZ conceived the study, participated in the methodology development, and helped to draft the manuscript. All authors read and approved the final manuscript.
